# O3-5 Family Health Climate, parental outcome expectations and physical activity of children in primary-school age

**DOI:** 10.1093/eurpub/ckac094.021

**Published:** 2022-08-29

**Authors:** Alexandra Ziegeldorf, Christina Niermann, Andreas Speer, Hagen Wulff, Heike Streicher, Petra Wagner

**Affiliations:** 1 University Leipzig, Institue for Exercise and Public Health, Leipzig, Germany; 2 University of Konstanz, Department of Sport Science, Konstanz, Germany

**Keywords:** physical activity, family health climate, outcome expectations, primary-school age

## Abstract

**Introduction:**

Children's health related behaviors, such as physical activity develop are maintained in the family environment. A large amount of studies examined different family influences on children's health behaviors. It has been shown that the Family Health Climate (FHC) (Niermann et al., 2015), and parents' general beliefs and expectations regarding children's PA are related to children's behaviour (Fredricks & Eccles, 2004). However, there is a lack of studies that examined changes in these variables over time and their relation to changes in children's physical activity. The aim of this study was to investigate a) if FHC and outcome expectations change from first to fourth grade in primary school and b) how they are related to decrease or increase of children's physical activity.

**Methods:**

380 consulted parents of primary-school age children (46,6 % male) continuously answered questions about their PA-related outcome expectations, FHC (predictor) and daily PA (outcome) of their children over four school years, starting 2014 with first grade (t1), third grade (t2), fourth grade (t3) in Leipzig, Germany. Repeated measures ANOVA were used to analyse changes over time.

**Results:**

Analysis showed no significant difference for FHC (F(1.96, 743.27) = 1.92, p =.15) an for parent's PA-related outcome expectations (F(1.98, 749.58) = 0.097, p = .91). Children's daily PA decrease significant over time (t1 to t3) (.04, 95%-CI[0.004, 0.343], p = .043).

**Conclusion:**

The results indicate stability of the FHC and parents' outcome expectations during primary school. In contrast, physical activity decreases over time, which is in line with previous studies. In further analyses we will examine how FHC and parents' outcome parents outcome expectations are related to changes in physical activity. In addition, future analyses should be performed gender-specific.

